# Densification and Mechanical Properties of Monolithic B_4_C and In-Situ Formed TiB_2_ Containing B_4_C–TiB_2_: Effects of Particle Size and Impurity

**DOI:** 10.3390/ma19132820

**Published:** 2026-07-02

**Authors:** İlker Solakoğlu, Furkan Buluç, Bahadır Tunaboylu, Servet Turan

**Affiliations:** 1 Department of Metallurgical and Materials Engineering, Faculty of Engineering, Marmara University, 34854 Istanbul, Türkiye; bahadir.tunaboylu@marmara.edu.tr; 2 TRBOR Boron Technologies Inc., 34906 Istanbul, Türkiye; 3 Ceramic Research Center, 26005 Eskişehir, Türkiye; afurkanbuluc@gmail.com (F.B.); sturan@eskisehir.edu.tr (S.T.); 4 Deputy Vice Chancellor Office of Graduate Research, Sultan Qaboos University, Al Khoud, Muscat P.O. Box 17, Oman; 5 Department of Materials Science and Engineering, Faculty of Engineering, Eskisehir Technical University, 26555 Eskişehir, Türkiye

**Keywords:** boron carbide (B_4_C), titanium carbide (TiC), particle size, purity, spark plasma sintering (SPS), mechanical properties, microstructure

## Abstract

This study investigates the effects of particle size, powder purity, and in situ TiB_2_ formation on the densification behavior and mechanical properties of B_4_C-based ceramics fabricated via spark plasma sintering (SPS). The results demonstrate that particle size is the dominant parameter controlling densification and microstructural evolution. An intermediate particle size (F1200) yields the highest relative density (98.41%) and superior mechanical properties, including a hardness of 33.34 GPa and fracture toughness of 4.63 MPa·m^1/2^, by providing an optimal balance between diffusion kinetics and particle packing. The in situ formed TiB_2_ significantly enhances fracture toughness compared to monolithic B_4_C. Among the composite samples, the medium particle size containing BT1200 exhibits the best overall combination of properties, with a relative density of 98.00%, hardness of 25.40 GPa, and fracture toughness of 5.18 MPa·m^1/2^. The highest fracture toughness is achieved in the largest particle size containing BT325 (6.29 MPa·m^1/2^), confirming the effectiveness of TiB_2_ combined with large particle size as a toughening phase. Although higher powder purity contributes to improved phase homogeneity, its influence on densification and mechanical performance is secondary compared to particle size and pore evolution. Excessive particle refinement leads to residual porosity, limiting full densification. Overall, optimizing particle size together with controlled in situ TiB_2_ formation is essential for achieving high-performance B_4_C-based ceramics and composites. These findings provide a practical framework for tailoring microstructure and mechanical properties in advanced ultra-hard ceramic systems.

## 1. Introduction

Boron carbide (B_4_C) is one of the most important structural ceramics due to its exceptional combination of low density, high hardness, chemical stability, and neutron absorption capability. Although the stoichiometric composition of boron carbide was defined as B_4_C in 1934, the material exhibits a broad homogeneity range with varying B/C atomic ratios. Nevertheless, the term B_4_C is conventionally used to describe this compound. As a strongly covalent non-oxide ceramic, B_4_C possesses a high melting temperature (~2427 °C), low density (~2.52 g/cm^3^), and extremely high hardness (~29 GPa), surpassed only by diamond and cubic boron nitride. In addition, its excellent wear resistance, chemical inertness, and high neutron absorption cross section (~600 barns) make it highly attractive for advanced engineering applications [1,2]. Consequently, B_4_C has been extensively used in lightweight armor systems, wear-resistant components, cutting tools, sandblasting nozzles, and neutron shielding materials for nuclear reactors [3,4,5,6].

Despite these superior properties, the densification of monolithic B_4_C remains highly challenging because of its strong covalent bonding, low self-diffusion coefficient, surface oxide impurities (primarily B_2_O_3_), and high melting point [7]. Conventional pressureless sintering and hot pressing methods generally require very high temperatures to achieve sufficient densification, which often leads to exaggerated grain growth and deterioration of mechanical performance. Numerous studies have therefore focused on the use of sintering additives and secondary reinforcement phases to improve densification behavior and fracture toughness. However, although additive-assisted approaches may lower the sintering temperature and improve toughness, they frequently cause reductions in hardness and elastic properties [8,9]. As a result, achieving a simultaneous combination of high hardness, high density, and improved fracture toughness in B_4_C-based ceramics remains a major challenge.

Among various reinforcement systems, TiB_2_ has attracted considerable attention due to its high hardness, high elastic modulus, excellent electrical conductivity, and thermodynamic compatibility with B_4_C. In recent years, significant progress has been achieved in the development of B_4_C–TiB_2_ composites, particularly through in situ reaction routes. Compared with externally added TiB_2_ particles, in situ formed TiB_2_ phases generally exhibit finer particle size, more homogeneous distribution, and stronger interfacial bonding with the B_4_C matrix. Previous studies demonstrated that the interconnected conductive TiB_2_ phase located along B_4_C grain boundaries significantly improved the mechanical properties of B_4_C–TiB_2_ composites, yielding fracture toughness values up to 4.38 MPa·m^1/2^ for composites containing 29.8 vol% TiB_2_ [10]. Similarly, optimized B_4_C–15 vol% TiB_2_ composites prepared using controlled B_4_C and TiC powder sizes achieved a relative density of 99.50%, hardness of 31.84 GPa, flexural strength of 780 MPa, and fracture toughness of 5.77 MPa·m^1/2^ [11]. In another study, TiO_2_-added B_4_C-based composites sintered at 1750 °C for 10 min exhibited improved densification and fracture toughness values reaching 5.08 MPa·m^1/2^ [12].

The improved performance of in situ B_4_C–TiB_2_ composites is mainly associated with the formation of a three-dimensional interconnected TiB_2_ network structure within the B_4_C matrix. The morphology and distribution of this TiB_2_ network can be effectively controlled by adjusting the particle sizes of the starting B_4_C and Ti-containing powders. Fine TiB_2_ particles formed during the in situ reaction promote grain boundary diffusion, reduce the activation energy for sintering, and facilitate densification at relatively lower temperatures [11,13]. Furthermore, the in situ formed TiB_2_ phase contributes to toughening through several mechanisms, including crack deflection, crack bridging, microcrack formation, and residual-stress toughening caused by the thermal expansion mismatch between B_4_C and TiB_2_ [14,15]. However, excessive coarsening or agglomeration of TiB_2_ particles may generate localized thermal stresses and negatively affect mechanical reliability.

**Figure 1:**
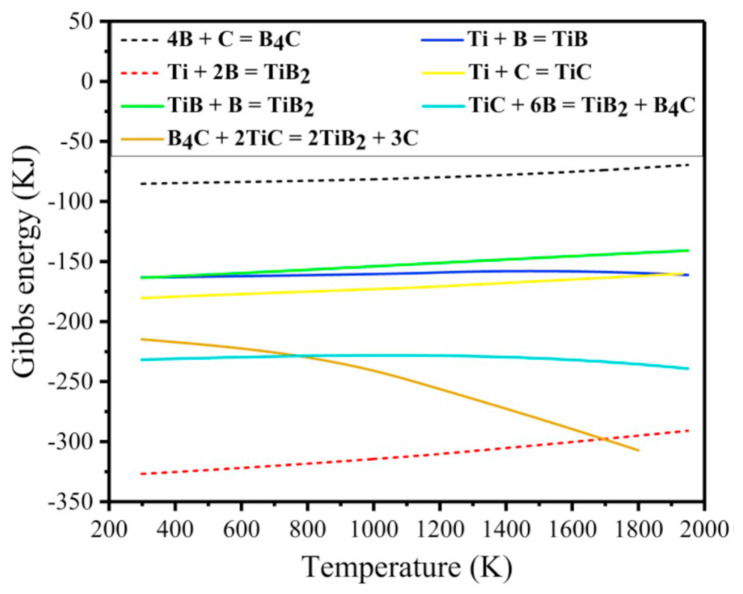
Gibbs free energy curves for reaction temperature [16].

Based on the recent literature, it is evident that the microstructural characteristics of in situ formed TiB_2_ phases, particularly their size, morphology, and distribution, play a critical role in determining the final densification behavior and mechanical performance of B_4_C-based composites. Therefore, further investigation of powder size effects and microstructural evolution is necessary to optimize the balance between hardness, densification, and fracture toughness in B_4_C–TiB_2_ ceramic systems. Thermodynamic Gibbs free energy provides an effective way to examine the reaction sequence and the formation of products in the sintered sample with increasing temperature. The Gibbs free energy curves as a function of reaction temperature are shown in Figure 1 [16].

As seen in the thermodynamic analysis in Figure 1, the Gibbs free energy of Reaction (1),

(1) $$B_{4}C+2TiC\to 2TiB_{2}+3C$$

is already highly negative at low temperatures (ΔG ≈ −220 kJ/mol at 400 K) and further decreases to approximately −310 kJ/mol at 1800 K, indicating a strong thermodynamic driving force for the in situ formation of TiB_2_ during Spark plasma sintering (SPS). The SPS method has been widely employed for the densification of ceramic powders. Compared to conventional sintering techniques, SPS offers shorter densification times, lower sintering temperatures, and higher densification levels, depending on parameters such as pulse duration, applied pressure, and powder characteristics [15,17]. The SPS method has been used for the densification of both monolithic B_4_C and B_4_C-based composite ceramics [18,19,20,21].

B_4_C–TiB_2_ composites have been synthesized via in situ reactions involving TiO_2_, carbon, and B_4_C, as described by Reaction (2) [22,23,24,25,26,27,28].

(2) $$B_{4}C+2TiO_{2}+3C\to 2TiB_{2}+4CO$$

Generally, TiB_2_ formation can also be achieved through the in situ reaction between TiC and B_4_C, as described by Reactions (3) [29]

(3) $$B_{4}C+2TiC\to 2TiB_{2}+3C$$

In this study, SPS was employed to process both monolithic B_4_C powders and TiC–B_4_C powder mixtures (33 wt.% TiC and 67 wt.% B_4_C). The influence of B_4_C particle size and powder purity on densification behavior, microstructural evolution, and mechanical properties was systematically investigated. For the composite samples, the TiB_2_ phase was formed in situ via the reaction between TiC and B_4_C during the SPS process.

## 2. Materials and Methods

### 2.1. Powder Preparation and Spark Plasma Sintering

Commercial B_4_C and TiC powders were used as starting materials. The technical specifications provided by the suppliers are listed in Table 1. Two experimental series were designed, each consisting of three compositions with a batch weight of 100 g.

In the first series, monolithic B_4_C powders with particle size grades of F325, F1200, and F2000 were used. In the second series, B_4_C powders of the same grades were mixed with 33 wt.% TiC. The sample designations and compositions are summarized in Table 2.

**Figure 2:**
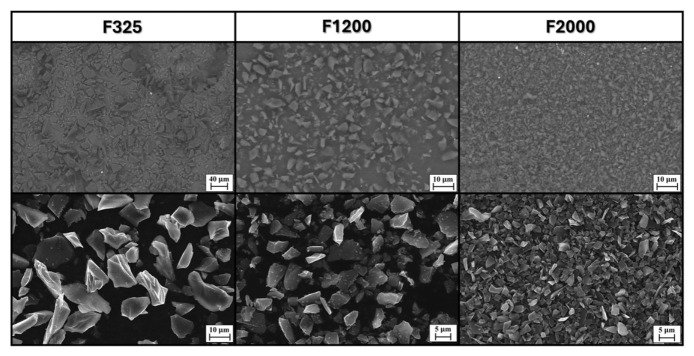
As-received Powder Morphology Analysis.

All powder mixtures were dispersed in 99.8% isopropanol (~30 vol% solids) and homogenized using a planetary ball mill (Fritsch Pulverisette, Idar-Oberstein, Germany), equipped with Si_3_N_4_ jars and balls. For composite powders, TiC was initially pre-ground at 350 rpm for 30 min to break up soft agglomerates and ensure homogeneous distribution in the mixture. Subsequently, B_4_C powder was added and the mixture was milled for an additional 60 min under identical conditions. Particle size analysis results of the as-received and milled powders are presented in Table 3, and the morphology analysis of the as-received powders is shown in Figure 2.

After milling, the slurries were dried using a rotary evaporator (Heidolph, Schwabach, Germany) at 55 °C, 45 rpm, and 195 mbar. The resulting powders were then oven-dried at 80 °C for 24 h and sieved through a 250-mesh screen to reduce agglomeration and improve flowability.

The prepared powders were loaded into a graphite die with an outer diameter of 40 mm and an inner diameter of 21.30–21.35 mm. Prior to sintering, the powders were pre-compacted at 20 MPa.

SPS was carried out using an FCT HPD 25 system under vacuum at a uniaxial pressure of 40 MPa. The heating rate was fixed at 100 °C/min, and temperature was monitored by an optical pyrometer focused on the graphite punch.

After sintering, the specimens were removed from the chamber, cleaned of residual graphite paper, and ground to obtain flat and parallel surfaces for subsequent characterization.

### 2.2. Characterization and Testing Methods

Phase identification was performed by X-ray diffraction (XRD) using a Rigaku, MiniFlex 600 diffractometer (Tokya, Japan)over a 2θ range of 20–70°, operating at 40 kV and 15 mA with a scanning rate of 1° min^−1^ and step size of 0.02°. Rietveld analysis was performed using Maud version 2.7 software.

For microstructural examination, the sintered samples were prepared by standard metallographic grinding and polishing procedures using diamond abrasives down to 1 μm finish. Surface morphology and phase distribution were analyzed using a scanning electron microscope equipped with BSE and EDS detectors.

Bulk density was measured by the Archimedes method in accordance with ASTM International ASTM C20 [30]. Relative density values were calculated using the theoretical densities of B_4_C (2.52 g/cm^3^) and TiC (4.93 g/cm^3^), applying the rule of mixtures for composite samples [29,31].

Vickers hardness was measured using an EMCOTEST Microhardness Tester (EMCO-TEST Prüfmaschinen GmbH, Kuchl, Austria). Microhardness measurements were taken by creating indentations under a load of 9.8 N and a holding time of 15 s. At least five measurements were taken for each sample and the average value was calculated [32].

Fracture toughness was estimated from indentation cracks using the Niihara approach, which is described in the literature, after applying a load of 27.6 N for 15 s, using Vickers indentation measurements [33].

Elastic modulus was determined by ultrasonic pulse velocity measurements based on the procedure described in Patent EP1788386A2 [34]. Sample thicknesses were measured using a micrometer, and the appropriate couplant was used.

## 3. Results

### 3.1. Densification Process and Microstructural Evaluation

**Figure 3:**
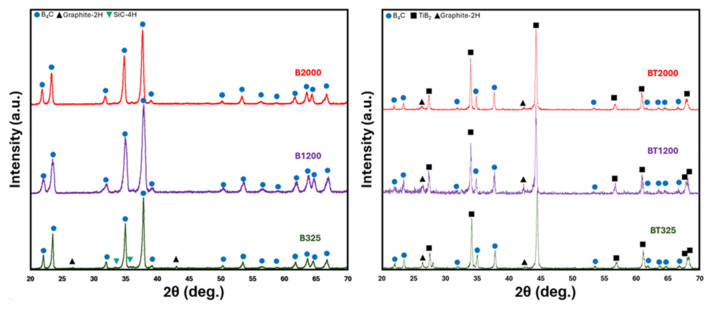
XRD patterns of monolithic B_4_C and B_4_C–TiC composite samples.

For the monolithic samples, boron carbide (B_4_C) is identified as the dominant crystalline phase in all compositions (Figure 3).

**Figure 4:**
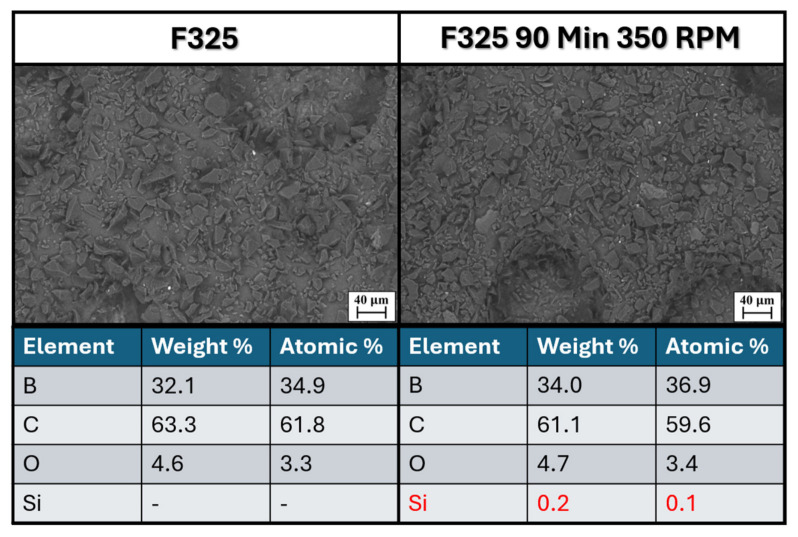
Impurity analysis of milled powder by EDS. The red regions indicate the distribution of Si after the milling process.

The main diffraction peaks located at 2θ ≈ 23°, 24°, 34°, and 38° are consistent with the standard rhombohedral B_4_C structure. Additionally, weak 26° diffraction peaks corresponding to graphite and hexagonal SiC were detected; the SiC phase visible on XRD is formed from the reaction of residual carbon and Si_3_N_4_ resulting from powder processing. The reason for the observation of the SiC phase in the B325 B_4_C sample only is determined to be the large size and sharp edges of the F325 class B_4_C powder particles, as shown in Figure 4. No detectable amount of SiC phase was identified in F1200 and F2000 powders milled under the same conditions.

For the composite samples, diffraction peaks associated with TiB_2_ were also detected, confirming that in situ reaction occurred between B_4_C and TiC during the SPS process. For B_4_C–TiC composite samples, XRD patterns show B_4_C and TiB_2_ as the dominant phases. No TiC peaks were detected, confirming that TiC was completely consumed during the process. Graphite formation was also observed in the diffraction patterns.

Quantitative phase analysis was performed using the Rietveld refinement method based on the XRD patterns, and the results are presented in Table 4. Results showed that the TiB_2_ content increased from approximately 37.4% in sample B325 to 41% in sample B2000, while the B_4_C content decreased. Graphite-2H (~7%) was detected in all samples (Table 4). This trend suggests that finer B_4_C starting powders enhance TiB_2_ formation by promoting the interface reaction between B_4_C and TiC during SPS. Furthermore, when the SiC 4H phase was included in the calculation, it was determined that this phase was present only in small amounts in the B325 sample.

**Figure 5:**
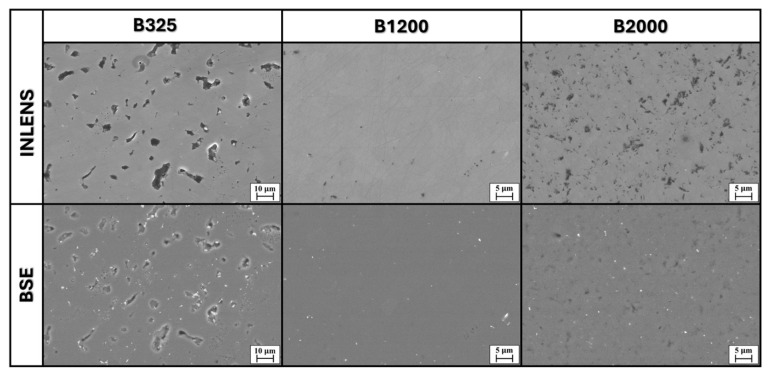
BSE and INLENS images of polished surface B325, B1200 and B2000.

BSE and INLENS images of polished surfaces showing that the microstructures are relatively homogeneous in all specimens are presented below in Figure 5.

The average grain sizes calculated using the ImageJ program IJ 1.46r on the images shown in Figure 5 are approximately 15–25 µm for B325, 4–10 µm for B1200, and 2–6 µm for B2000. In the B325 sample, SiC grains resulting from the milling process can be observed with white contrast. The presence of large grains and large pore clusters between these grains was determined in the B325 sample. However, the B1200 sample showed very little porosity and carbon inclusions. EDS analysis of the B1200 BSE image revealed that the small white grains are SiC. XRD analysis did not show a peak in the region where this phase was present due to the very low amount of SiC. Density measurements and microstructure analysis showed a higher concentration of pores and carbon inclusions compared to B1200, while the B2000 material also showed a higher number of smaller pores. The in-lens technique is particularly suitable for separating carbon, porosity, and B_4_C.

**Figure 6:**
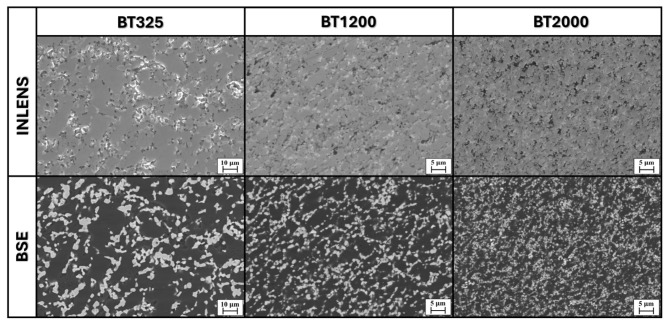
BSE and INLENS images of polished surfaces of BT325, BT1200, BT2000.

The measured relative densities of the monolithic samples were 96.0% (B325), 98.4% (B1200), and 98.0% (B2000), respectively. Figure 6 presents the BSE and INLENS SEM images of the composite samples. The dark regions correspond to the B_4_C matrix, while the bright regions represent the TiB_2_ phase in BSE images.

When the BT325 sample was examined, it was determined that it had a finer microstructure but a higher densification compared to the B325 sample. One reason for this is that TiC inhibits the growth of B_4_C grain size as it transforms into TiB_2_ and graphite in the structure. Another finding is that the carbons cluster together in the BT325 sample, and the resulting TiB_2_ grains are much larger than those in BT1200 and BT2000. In the BT1200 sample, the TiB_2_ grains and associated graphite are homogeneously distributed. The densification of this sample is microstructurally similar to B1200. It was determined that the TiB_2_ grains are smaller than in BT325 but larger than in BT2000. In the BT2000 sample, it was determined that the densification is higher in the regions where the B_4_C grains are attached to each other compared to the TiB_2_ regions. As expected, the finest microstructure was obtained in BT2000. The average grain sizes are approximately 10–15 µm for BT325, 2–5 µm for BT1200, and 1–2 µm for BT2000. The corresponding relative densities are 97.7%, 98.0%, and 96.7%, respectively.

**Figure 7:**
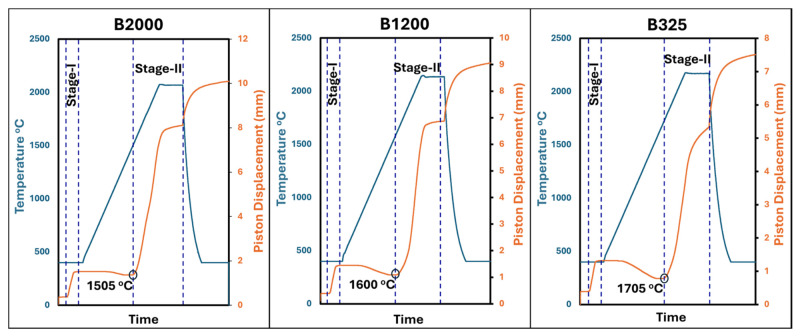
Densification behavior of monolithic boron carbide samples during SPS. The vertical blue dashed lines represent the boundaries between the processing stages and key transition points during the cycle.

**Figure 8:**
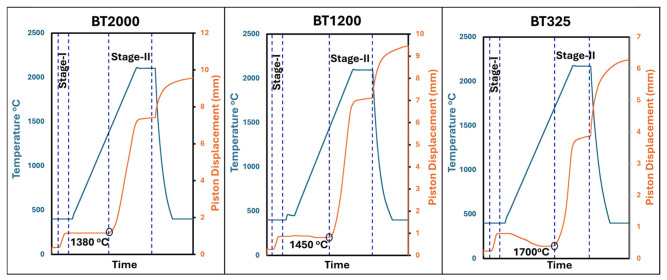
Densification behavior of composite boron carbide samples during SPS. The vertical blue dashed lines represent the boundaries between the processing stages and key transition points during the cycle.

The densification behavior during SPS is shown in Figure 7 and Figure 8.

In the SPS graphs, stage-1 represents the point where the pressure is increased to 40 MPa. Examining the graphs, it is observed that the densification of the monolithic B_4_C material, which has a low grain size, begins at 1505 °C, B1200 at 1600 °C, and B325 at 1705 °C. When TiB_2_ is added, the densification onset temperature decreases by 120 °C and 150 °C for BT2000 and BT1200, respectively. However, this temperature remains unchanged in the BT325 sample. Given the very high grain size of B325, and consequently the lower reaction rate of the TiC grains, the unchanged densification onset in B325 and BT325 is an expected result. Furthermore, even though they start densifying at the same temperature, the curve shows that BT325 material densifies faster, as evidenced by the flattening of the curve towards the middle of the sintering process. Among the composite samples, the highest relative density is observed for BT1200 (98.0%), while the lowest value is measured for BT2000 (96.7%).

### 3.2. Mechanical Properties

The mechanical properties of monolithic B_4_C and B_4_C–TiC composite samples are summarized in Table 5. The results show that mechanical behavior depends largely not only on relative density but also on microstructural homogeneity, grain sizes, and the distribution and size of secondary phases within the composite.

Among the monolithic B_4_C samples, B1200 exhibited the best overall mechanical performance, achieving the highest relative density (98.4%), Young’s modulus (463.4 GPa), hardness (33.34 GPa), and fracture toughness (4.63 MPa·m^1/2^). As shown in Figure 5, B1200 has better performance than B235 due to its smaller grains and higher densification compared to B2000, as well as its larger secondary phase grains. Although all samples can be sintered with SPS, the B1200 grain size and post-sintering microstructure have been determined as the optimum point. In the literature, it is stated that samples consolidated at 1900 °C under 40 MPa have a relative density of 93.87% and a hardness of 26.40 GPa [21]. In addition, B_4_C powders with an average grain size of 1.47 μm sintered with SPS at 1850 °C under 50 MPa with a heating rate of 100 °C/min and a holding time of 10 min reached a fracture toughness of 3.21 MPa·m^1/2^ and a Vickers hardness of 33.5 GPa [35]. Similarly, increasing the applied pressure to 80 MPa at 1700 °C yielded a relative density of 99.70%, a hardness of 37.50 GPa, and a fracture toughness of 4.7 MPa·m^1/2^ [36]. The hardness and fracture toughness values obtained for the B1200 specimen in this study are comparable to previously reported literature data, despite differences in sintering parameters and processing conditions. In contrast, B325 exhibited lower mechanical properties; its relative density was 96.0%, its hardness was 30.2 GPa, and its Young’s modulus was 445.75 GPa. The coarser initial powder size negatively affected densification, limiting diffusion and neck growth during sintering. SEM observations in Figure 5 revealed relatively large closed pores and excessively large grain growths. Pores can reduce the material’s elastic modulus as well as its hardness. However, these decreases are less pronounced in fracture toughness because these voids can lead to fractures in an infinite loop. Although B2000 reaches a relatively high density (98.0%), its Young’s modulus drops to 422.2 GPa. As shown in Figure 5, the microstructure contains fine closed voids, which may be related to the aggregation of fine starting powders. Such aggregation can create closed porosity, leading to a decrease in elastic modulus despite the seemingly high density.

The mechanical properties of the composite samples differed significantly from those of monolithic B_4_C due to the formation of TiB_2_ reinforcement phases, as confirmed by XRD analysis. All composite specimens exhibited markedly higher fracture toughness than monolithic B_4_C, indicating the beneficial toughening effect of the in situ formed TiB_2_. Similar improvements in toughness have been reported in previous studies, where the interconnected conductive TiB_2_ phase located at B_4_C grain boundaries significantly enhanced the mechanical performance of B_4_C–TiB_2_ ceramic composites [10,11].

**Figure 9:**
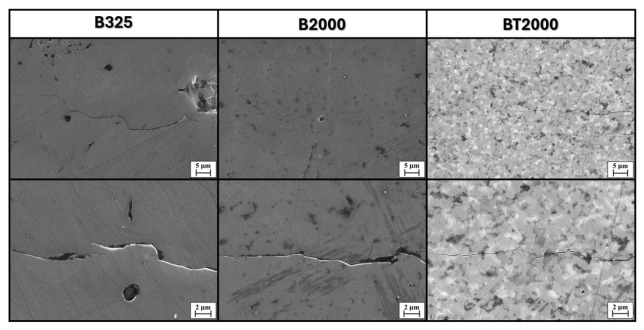
Microstructures of cracks upon Vickers indentation on B325, B2000, BT2000.

Among the existing composites, BT325 exhibited the highest fracture toughness (6.29 MPa·m^1/2^). As shown in Figure 6, the microstructure contained relatively large and interconnected bright TiB_2_-rich regions dispersed in the B_4_C matrix. The grown TiB_2_ secondary phase enhanced crack deflection, crack bridging, and crack branching mechanisms, as shown in Figure 9, thus increasing fracture toughness. The superior toughness obtained in this study clearly demonstrates the effect of the starting powder grain size and impurities arising from process residues on fracture toughness.

BT1200 exhibited the most balanced mechanical performance among the composites, combining the highest relative density (98.0%), a hardness of 25.40 GPa, and a fracture toughness of 5.18 MPa·m^1/2^. As shown in Figure 6, this homogeneous microstructure likely improved stress transfer between the matrix and reinforcement while maintaining structural integrity. Similar behavior has also been reported inTiO_2_-added B_4_C-based composites sintered at 1750 °C for 10 min, where increased densification resulted in a relative density of 98.5% and a fracture toughness of 5.77 MPa·m^1/2^ [11]. BT2000 exhibited the lowest relative density (96.7%) and correspondingly lower fracture toughness (5.02 MPa·m^1/2^) among the composites. As shown in Figure 6, the microstructure was determined to consist of finely structured TiB_2_ and B_4_C grains with increasing closed porosity. The TiB_2_ grains prevented the densification of B_4_C, thus reducing the hardness of the composite (25.64 GPa) compared to monolithic B_4_C. In B325 material, Vickers indentation fracture toughness occurs due to large grains, and crack bridging is also observed. Similarly, fracture deflection was determined in B2000 material. Fracture coincides with porosity and deflections from there. Specifically, it was determined that the fracture propagation around the TiB_2_ phases and the fracture deflection in these regions were greater in BT2000 compared to monolithic B_4_C B2000 (Figure 9).

Overall, the results show that optimum mechanical performance is achieved when high density is combined with a homogeneous microstructure and well-dispersed TiB_2_ reinforcement. Therefore, the medium powder size condition (F1200) provided the optimal balance between density, stiffness, and toughness in both monolithic and composite systems. The obtained mechanical properties are similar to or higher compared to many B_4_C–TiB_2_ composites previously reported in the literature, demonstrating the effectiveness of the current processing approach in shaping the microstructure and improving fracture resistance.

## 4. Discussion

The XRD results confirm that the formation of TiB_2_ in composite samples demonstrates the successful in situ reaction between B_4_C and TiC during SPS. The complete disappearance of TiC peaks further supports this transformation. The higher intensity of TiB_2_ diffraction peaks compared to B_4_C can be attributed to differences in atomic scattering factors.

The densification behavior was strongly influenced by powder particle size, with decreasing particle size promoting enhanced diffusion and earlier shrinkage onset during SPS. Although the densification in sample BT325 started at the same temperature as in sample B325, it was determined by SPS piston movements that it was better towards the peak temperature because of in-situ reactions between B_4_C and TiC. However, the highest relative density was achieved for the B1200 sample rather than the finest powder condition, indicating that excessive particle size reduction does not necessarily guarantee optimum densification. It has been determined that when the B_4_C grain size is very small, the TiB_2_ formed as a result of reactions with TiC negatively affects densification. This observation suggests that an intermediate particle size provides a more favorable balance between surface energy, particle packing efficiency, and pore elimination. Similar trends have been reported in previous B_4_C–TiB_2_ systems, where optimized powder size distribution resulted in superior densification and mechanical performance [11].

Furthermore, the absence of abnormal grain growth in all samples indicates that SPS effectively suppresses grain coarsening.

Among the composites, BT1200 exhibited the highest densification, consistent with the behavior of the monolithic B1200 specimen, suggesting an optimal balance between particle size and densification. In contrast, the slightly lower densification of BT2000 despite its finer grain size has been attributed to the diffusion hindrance of the fine TiB_2_ grains formed during sintering. This suggests that fine starting powders can accelerate densification kinetics in the early stages while simultaneously increasing the likelihood of pore confinement with the secondary phase formed as a result of reactions at high temperatures.

During sintering, TiB_2_ grains formed and grew within the B_4_C matrix, contributing significantly to the final composite microstructure. The distribution and morphology of TiB_2_ particles strongly affected fracture behavior and mechanical performance through crack deflection, crack bridging, and crack branching mechanisms [11,14]. Previous studies reported fracture toughness values of approximately 4.38 MPa·m^1/2^ for B_4_C–29.8 vol% TiB_2_ composites [10], while optimized in situ B_4_C–15 vol% TiB_2_ systems achieved fracture toughness values up to 5.77 MPa·m^1/2^ together with high hardness and density [11]. In the present study, the BT325 sample exhibited a higher fracture toughness of 6.29 MPa·m^1/2^, which can be seen from the fracture propagation microstructures as effectively increasing the fracture toughness obtained under the current SPS conditions. However, the results also show that coarse or clustered TiB_2_ regions can reduce hardness, while excessive thinning can reduce crack bridging ability. Therefore, controlling the TiB_2_ particle size and distribution remains one of the most important unresolved problems in the optimization of B_4_C–TiB_2_ composites.

The SPS densification behavior showed a strong dependence on the initial powder size. As shown in Figure 7 and Figure 8, decreasing powder size systematically shifted both the shrinkage onset and the main densification phase to lower temperatures. B2000 showed the earliest shrinkage onset, while the maximum relative density was obtained for B1200 (98.41%). Similarly, B_4_C–TiC composites exhibited earlier shrinkage onset and improved densification compared to monolithic samples, highlighting the beneficial role of Ti-containing additives during SPS [37]. However, despite the accelerated densification kinetics observed in BT2000, its relatively low density (96.67%) indicates that rapid shrinkage alone is not sufficient to achieve full densification. Even though the initial portions of the densification curve of samples B325 and BT325 were the same, BT325 was determined to densify faster. These findings indicate that accurate results can be obtained by simultaneously optimizing densification kinetics, pore formation, and microstructural homogeneity.

From a mechanical perspective, the behavior of both monolithic and composite samples was primarily determined by relative density and particle size, while the effect of powder purity was secondary. Mechanical properties showed a strong correlation with relative density, as increased density reduced porosity and improved interparticle bonding. Among monolithic samples, B1200 had the highest relative density (98.41%) and the highest hardness (33.34 GPa) and Young’s modulus (463.43 GPa). In contrast, the relatively lower density of B325 resulted in reduced hardness and rigidity due to the presence of residual pores acting as stress concentrators. A similar trend was observed in composite systems; BT1200 exhibited the most stable mechanical performance thanks to its high density and homogeneous TiB_2_ distribution. Although BT325 showed the highest fracture toughness (6.29 MPa·m^1/2^), its lower hardness was associated with larger TiB_2_-rich regions and slightly lower density. The high fracture toughness is due to the greater presence of fracture toughness mechanisms along the fracture, as can be seen in the fracture microstructure. Furthermore, the low density and grain size of BT2000 negatively impacted its fracture toughness despite its relatively high hardness; this is due to the lower activity of active fracture toughness mechanisms resulting from the almost straight progression of the fracture due to its small grain size. These findings demonstrate that optimum mechanical performance in B_4_C-based ceramics can only be achieved through the simultaneous control of densification, pore elimination, and reinforcement distribution. Among these, BT325 exhibited the highest fracture toughness, while BT1200 provided the most balanced combination of density, hardness, and toughness.

Furthermore, compared to the SPS-processed sample reported in [21] (97.1% purity, d_50_ = 1.2 µm, sintered at 1900 °C), which exhibited 93.87% relative density and 26.4 GPa hardness, the B2000 sample sintered at 2110 °C displayed significantly improved densification and competitive hardness values. This improvement can be attributed to differences in powder properties and optimized SPS processing conditions. However, the persistence of residual porosity in fine powder systems indicates that further optimization of the heating profile, holding time, and particle distribution is still necessary. Overall, the results demonstrate that the increasingly fine powder size and in-situ TiB_2_ formation synergistically enhance densification and mechanical performance during SPS processing. At the same time, the study reveals several remaining challenges, including residual pore confinement in fine powders, optimization of TiB_2_ distribution, and the need to simultaneously maximize hardness, density, and fracture toughness. These issues should be addressed in future studies to further improve the reliability and structural performance of B_4_C–TiB_2_ ceramic composites.

This study also demonstrated that using coarse powders like F325 instead of fine powders like F2000, and achieving positive results, can reduce the carbon footprint in the production of B_4_C composite ceramics.

## 5. Conclusions

This study highlights the critical role of particle size, powder purity, and in-situ TiB_2_ formation on the densification and mechanical performance of B_4_C-based ceramics processed by the SPS method. Among these parameters, particle size was determined to be the dominant factor, with the medium particle size (F1200) providing the highest densification and optimum mechanical properties by balancing diffusion kinetics. In-situ TiB_2_ formation significantly increased fracture toughness compared to monolithic B_4_C and confirmed its effectiveness as a toughness enhancement strategy in conjunction with particle size.

Among the composite samples, BT1200 exhibited the best overall combination of density, hardness, and toughness. While higher powder purity contributed to improved phase homogeneity and reduced impurity-related effects, its effect on densification and mechanical properties was found to be secondary compared to particle size and pore evolution. In contrast, excessive particle size refinement led to residual porosity in the composites.

Overall, the results demonstrate that optimizing particle size while maintaining adequate powder purity and ensuring controlled in-situ TiB_2_ formation is essential for obtaining high-performance B_4_C-based ceramics. These findings provide a practical framework for tailoring the microstructure and mechanical properties in advanced ultrahard ceramic systems.

Furthermore, the successful application of ground powders like F325, which have a lower carbon footprint, in composites removes barriers to using coarse powders.

## Figures and Tables

**Table 1:** Technical Specifications of B_4_C and TiC Raw Material Powders.

Powder Code	Purity (%)	Particle Size (μm)	Supplier	Country
B_4_C (F2000 grade)	≥98	1	CRS Chemicals	Los Angeles, CA, USA
B_4_C (F1200 grade)	≥95	3	TRBOR Bor Teknolojileri A.Ş	Istanbul, Türkiye
B_4_C (F325 grade)	≥97	16	TRBOR Bor Teknolojileri A.Ş	Istanbul, Türkiye
TiC	≥99	1–5	Nanokar Kimyevi Maddeler San. ve Tic Ltd.	Istanbul, Türkiye

**Table 2:** Sample designations of compositions.

Sample Designation	Composition (%)	Ball Milling Conditions	Final Phases
B325	100 B_4_C	90 min 350 RPM	B_4_C
B1200	100 B_4_C		B_4_C
B2000	100 B_4_C		B_4_C
BT325	67 B_4_C, 33 TiC	TiC milled for 30 min at 350 rpm, followed by mixture milling for 60 min at 350 rpm	B_4_C + TiB_2_ + C
BT1200	67 B_4_C, 33 TiC		B_4_C + TiB_2_ + C
BT2000	67 B_4_C, 33 TiC		B_4_C + TiB_2_ + C

**Table 3:** Particle Size Analysis of As-received and Milled Powders.

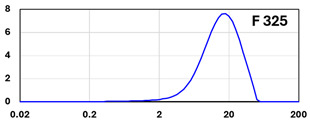	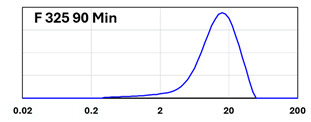				
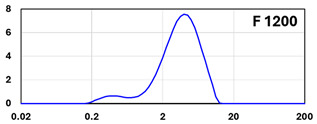	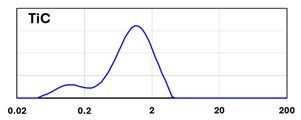				
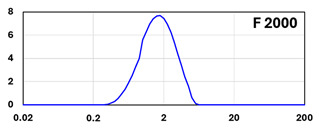	**Powder Code**	**Particle Size Distribution (μm)**			
		D10	D50	D90	D100
	**B_4_C (F2000 grade)**	0.77	1.69	3.37	6.29
	**B_4_C (F1200 grade)**	1.22	3.41	6.80	11.55
	**B_4_C (F325 grade)**	6.83	16.49	32.82	52.75
	**B_4_C (F325 grade) 90 min 350 RPM**	4.98	13.44	26.91	45.19
	**TiC**	0.17	1.04	2.33	4.48

**Table 4:** Crystalline phase as calculated by Rietveld analysis from the XRD patterns (Figure 3) of monolithic B_4_C and B_4_C-composites.

Sample Designation	Crystalline Phases (wt.%)			
	B_4_C	Graphite-2H	SiC 4H	TiB_2_
B2000	100	-	-	-
B1200	100	-	-	-
B325	99.2	0.2	0.6	-
BT2000	52.2	7.1	-	40.7
BT1200	53.6	6.5	-	39.9
BT325	56.3	6.3	-	37.4

**Table 5:** Mechanical properties of monolithic and composite samples.

Specimen	RD (%)	Bulk Density (g/cm^3^)	Young Modulus (GPa)	Hardness (GPa)	Indentation Fracture Toughness Kıc (MPa.m^1/2^)
B325	96.0	2.42	446	30.2	4.5
B1200	98.4	2.48	463	33.3	4.6
B2000	98.0	2.47	422	32.2	4.6
BT325	97.7	2.93	376	21.6	6.3
BT1200	98.0	2.94	336	25.4	5.2
BT2000	96.7	2.90	366	25.6	5.0

## Data Availability

The original contributions presented in this study are included in the article. Further inquiries can be directed to the corresponding author.

## Abbreviations

B_4_C	Boron carbide
PWRs	Pressurized water reactors
LMFBRs	Liquid metal fast breeder reactors
TiB_2_	Titanium diboride
TiC	Titanium carbide
TiO_2_	Titanium oxide
SPS	Spark Plasma Sintering

## References

[1] Sonber J.K., Murthy T.S.R.C., Subramanian C., Fotedar R.K., Hubli R.C., Suri A.K. (2013). Synthesis, Densification and Characterization of Boron Carbide. Trans. Indian Ceram. Soc..

[2] Thevenot F. (1990). Boron Carbide: A Comprehensive Review. J. Eur. Ceram. Soc..

[3] Levin L., Frage N., Dariel M.P. (2000). A novel approach for the preparation of B4C-based cermets. Int. J. Refract. Met. Hard Mater..

[4] Xie K.Y., Kuwelkar K., Haber R.A., LaSalvia J.C., Hemker K.J. (2016). Microstructural Characterization of a Commercial Hot-Pressed Boron Carbide Armor Plate. J. Am. Ceram. Soc..

[5] Toksoy M.F., Rafaniello W., Xie K.Y., Luoning M., Hemker K.J., Haber R.A. (2017). Densification and characterization of rapid carbothermal synthesized boron carbide. Int. J. Appl. Ceram. Technol..

[6] Gosset D., Colin M. (1991). Boron carbides of various compositions: An improved method for X-rays characterization. J. Nucl. Mater..

[7] Suri A.K., Subramanian C., Sonber J.K., Murthy T.S.R.C. (2010). Synthesis and Consolidation of Boron Carbide: A Review. Int. Mater. Rev..

[8] Twardowska A., Kowalski M. (2024). The Microstructure, Mechanical, and Friction-Wear Properties of Boron Carbide-Based Composites with TiB_2_ and SiC Formed In Situ by Reactive Spark Plasma Sintering. Materials.

[9] Roy T., Subramanian C., Suri A. (2006). Pressureless sintering of boron carbide. Ceram. Int..

[10] Ren D., Deng Q., Wang J., Yang J., Li Y., Shao J., Li M., Zhou J., Ran S., Du S. (2018). Synthesis and properties of conductive B_4_C ceramic composites with TiB_2_ grain network. J. Am. Ceram. Soc..

[11] Zhao J., Fang Z., Jin X., Wang D., Ding X., Ran S. (2023). B_4_C–TiB_2_ composite with modified microstructure and enhanced properties from optimal size coupling of raw powders. J. Am. Ceram. Soc..

[12] Du Z., Yi M., Chen S., Zhang J., Xiao G., Chen Z., Chen H., Xu C. (2024). Toughening mechanism of B_4_C/TiB_2_ ceramic materials by in situ reaction via spark plasma sintering. Int. J. Appl. Ceram. Technol..

[13] Skorokhod V.V. (2000). Processing Microstructure, and Mechanical Properties of B_4_C—TiB_2_ Particulate Sintered Composites I. Pressureless Sintering and Microstructure Evolution. Powder Metall. Met. Ceram..

[14] Skorokhod V.V. (2000). Processing, Microstructure, and Mechanical Properties of B_4_C—TiB_2_ Particulate Sintered Composites. Part II. Fracture and Mechanical Properties. Powder Metall. Met. Ceram..

[15] Huang S., Vanmeensel K., Malek O.J.A., Van der Biest O., Vleugels J. (2011). Microstructure and mechanical properties of pulsed electric current sintered B_4_C–TiB_2_ composites. Mater. Sci. Eng. A.

[16] Wang S., Yuan J., Han W., Yin Z. (2020). Microstructure and mechanical properties of B_4_C-TiB_2_ composite ceramic fabricated by reactive spark plasma sintering. Int. J. Refract. Met. Hard Mater..

[17] Hayun S., Kalabukhov S., Ezersky V., Dariel M.P., Frage N. (2010). Microstructural characterization of spark plasma sintered boron carbide ceramics. Ceram. Int..

[18] Liu L., Zhang Z., Jia X., Wang Q., Song Q., Li X., Cheng X. (2024). The effect of spark plasma sintering parameters on the densification and mechanical properties of TiB_2_-TiC-TiB composites. Int. J. Appl. Ceram. Technol..

[19] Grippi T., Torresani E., Maximenko A.L., Olevsky E.A. (2024). Spark plasma sintering of boron carbide (B_4_C): From characterisation to finite element modeling of sintering. J. Eur. Ceram. Soc..

[20] Hayun S., Paris V., Dariel M., Frage N., Zaretzky E. (2009). Static and dynamic mechanical properties of boron carbide processed by spark plasma sintering. J. Eur. Ceram. Soc..

[21] Zhang X., Zhang Z., Wen R., Wang G., Zhang X., Mu J., Che H., Wang W. (2018). Comparisons of the densification, microstructure and mechanical properties of boron carbide sintered by hot pressing and spark plasma sintering. Ceram. Int..

[22] Yamada S., Hirao K., Yamauchi Y., Kanzaki S. (2003). High strength B_4_C–TiB_2_ composites fabricated by reaction hot-pressing. J. Eur. Ceram. Soc..

[23] Khajehzadeh M., Ehsani N., Baharvandi H.R., Abdollahi A., Bahaaddini M., Tamadon A. (2020). Thermodynamical evaluation, microstructural characterization and mechanical properties of B_4_C–TiB_2_ nanocomposite produced by in-situ reaction of Nano-TiO. Ceram. Int..

[24] Huang S.G., Vanmeensel K., Van der Biest O., Vleugels J. (2011). In situ synthesis and densification of submicrometer-grained B_4_C–TiB_2_ composites by pulsed electric current sintering. J. Eur. Ceram. Soc..

[25] Švec P. (2025). Wear Resistance of B_4_C-TiB_2_ Ceramic Composite. Lubricants.

[26] Švec P., Gábrisová Z., Brusilová A. (2020). Reactive sintering of B_4_C-TiB_2_ composites from B_4_C and TiO_2_ Precursors. Process. Appl. Ceram..

[27] Li X., Chen B., Qiao J., Tang J., Sahin F.C., Yucel O. (2024). Low-temperature synthesis and sinterability of high-purity submicron TiB_2_ powder via microwave-assisted carbothermal reduction. J. Eur. Ceram. Soc..

[28] Skorokhod V., Krstic V.D. (2000). High strength-high toughness B_4_C-TiB_2_ composites. J. Mater. Sci. Lett..

[29] Wang Z., He Q., Ren S., Wang A., Wang W. (2024). Effects of TiC content on the microstructure and properties of B_4_C–TiB_2_–graphite composites by reactive hot pressing. J. Eur. Ceram. Soc..

[30] (2015). Standard Test Methods for Apparent Porosity, Water Absorption, Apparent Specific Gravity, and Bulk Density of Burned Refractory Brick and Shapes by Boiling Water.

[31] Baskut S., Ozer S.C., Turan S. (2022). The effects of in-situ formed phases on the microstructure, mechanical properties and electrical conductivity of spark plasma sintered B_4_C containing Y_2_O_3_. J. Eur. Ceram. Soc..

[32] Fırdous A., Rasool T., Dar G.N., Ahmad M.M. (2010). Micro-Mechanical Studies on Pure and Ni Doped ZnS Nanostructured Crystals. J. Optoelectron. Biomed. Mater..

[33] Liang K.M., Orange G., Fantozzi G. (1990). Evaluation by indentation of fracture toughness of ceramic materials. J. Mater. Sci..

[34] Buluc A.F., Celikkardes E., Turan S., Kim H.-J., Kim Y.-W. (2025). The effect of hot forging on the thermal and mechanical properties of spark plasma sintered SiC-TiB_2_-B_4_C Composites with CeO_2_ Addition. J. Eur. Ceram. Soc..

[35] Liu Y., Ge S., Huang Y., Huang Z., Zhang D. (2021). Influence of Sintering Process Conditions on Microstructural and Mechanical Properties of Boron Carbide Ceramics Synthesized by Spark Plasma Sintering. Materials.

[36] Ji W., Rehman S.S., Wang W., Wang H., Wang Y., Zhang J., Zhang F., Fu Z. (2015). Sintering boron carbide ceramics without grain growth by plastic deformation as the dominant densification mechanism. Sci. Rep..

[37] Sigl L.S. (1998). Processing and mechanical properties of boron carbide sintered with TiC. J. Eur. Ceram. Soc..

